# Bivalent SARS-CoV-2 spike immunization with non-replicative antibiotic resistance-free DNA vaccine induces immunity to multiple virus variants

**DOI:** 10.1038/s41598-025-23919-7

**Published:** 2025-11-14

**Authors:** Jaime Larraga, Pablo Nogales-Altozano, Laro Gomez-Marcos, Silvia Ruiz, Francisco Javier Loayza, Alicia Rivera-Rodríguez, Andrés Louloudes-Lázaro, Ana B. Carlon, Daniel Rodríguez-Martín, Ana Alonso, Verónica Martín, Pedro J. Alcolea, José M. Rojas, Vicente Larraga, Noemí Sevilla

**Affiliations:** 1 Centro de Investigación en Sanidad Animal, Instituto Nacional de Investigación y Tecnología Agraria y Alimentaria, Consejo Superior de Investigaciones Científicas (CISA-INIA-CSIC), Valdeolmos, Madrid, Spain; 2 Centro de Investigaciones Biológicas Margarita Salas, Consejo Superior de Investigaciones Científicas (CIB-CSIC), Madrid, Spain; 3 Universidad Autónoma de Madrid (UAM), Escuela de Doctorado, Madrid, Spain

**Keywords:** Needle-free injection system, DNA vaccine, Neutralization, Antibodies, Protection, SARS-CoV-2, Immunology, Microbiology

## Abstract

Severe Acute Respiratory Syndrome Coronavirus-2 (SARS-CoV-2), the cause of the COVID-19 pandemic, continues to evolve, with new variants still causing mortality in vulnerable populations. Developing vaccines that induce immunity against multiple variants and can be rapidly adapted is key to address future threats. In this study, we assessed the immunogenicity and protective efficacy of a non-replicative antibiotic resistance-free DNA vaccine (pPAL) expressing the stabilized spike proteins from the Wuhan and Omicron variants, as well as the conserved nucleoprotein. K18-hACE2 mice received a prime-boost-boost vaccination with three pPAL plasmids encoding the Wuhan spike, Omicron spike, or Wuhan nucleoprotein. The vaccine induced antibody responses against recombinant receptor-binding domains from both spike protein variants and promoted a Th1 bias immune response, as indicated by increased IgG2a/c levels compared to IgG1. Neutralization antibodies were generated against both SARS-CoV-2 variants. Furthermore, vaccinated mice were protected from a lethal challenge with the Wuhan isolate, showing reduced viral replication in target organs. This study presents a DNA vaccine platform capable of expressing multiple SARS-CoV-2 antigens and inducing robust immunity against different viral variants. This approach offers a promising and adaptable strategy for future SARS-CoV-2 vaccination efforts.

## Introduction

Vaccination successfully controlled the pandemic produced by SARS-CoV-2, which helped restore the global economy^1^. Nonetheless, variants from the original Wuhan virus have emerged throughout the pandemic as the virus mutated to adapt to the human host and escape immunity^2^. The emergence of these SARS-CoV-2 variants represents a major challenge that requires vaccines to be updated to protect vulnerable populations. Indeed, current vaccines have altered their formulation to incorporate the latest forms of the spike protein so that antibody responses are directed to the circulating strains of virus^3,4^. Because of the shifting antigenic landscape of SARS-CoV-2^5^, the design of multivalent vaccines that can protect against several virus strains is now highly sought after^6,7^.

DNA vaccine platforms can provide an answer to this issue as they can be rapidly modified to accommodate new antigens. Moreover, they can be engineered to express several pathogen antigens, which allow for rapid testing against new pathogen strains or new emerging threats. DNA vaccines are effective at inducing immunity and protecting against diseases and they have been successfully used in veterinary medicine^8,9^. DNA vaccines are also affordable and their production can be easily scaled-up. Finally, they are thermotolerant, which allow for their storage without relying on strict cold storage conditions. These features make DNA vaccines particularly attractive for vaccination programs in developing countries since production can rapidly and inexpensively shift towards the antigens of new variants.

To improve DNA vaccine immunogenicity, delivery methods need to be optimized to achieve adequate levels of antigen expression. This can be done using encapsulation of the nucleic acid material with charged polymers to promote transfection^10^, or through physical means such as electroporation to favor DNA uptake. In the present work, we used a needle-free injection system (NFIS) for antigen delivery that eliminates the potential hazard of contaminated needles for the handler. Furthermore, this inoculation strategy does not require costly DNA encapsulation that also increases the handling complexity of the DNA vaccine. This system is based on delivering the DNA vaccine as a high-pressure jet stream that penetrates the intramuscular, subcutaneous, or intradermal tissue depth, and is considered an alternative to electroporation, as it minimizes injection discomfort and increases the tissue area in which the vaccine is delivered, reaching the subcutaneous tissue that contains a high density of dendritic and other antigen presenting cells^11^.

One of the main limitations of DNA vaccines is the frequent inclusion of antibiotic resistance genes for plasmid selection during the manufacturing process, which raises safety concerns for clinical use. To address this, we developed the pPAL plasmid—a non-replicative, antibiotic resistance-free mammalian expression vector that enables selection without the use of antibiotics^12,13^. The pPAL plasmid uses the *Escherichia coli* chromosomal *fabI* gene, which encodes the enoyl-ACP reductase (FabI), as a selectable marker. This allows for triclosan-based selection, eliminating the need for antibiotic resistance genes and significantly improving the biosafety profile of the vector for regulatory approval.We previously demonstrated that a DNA vaccine based on this platform, expressing the spike and nucleoprotein of SARS-CoV-2, conferred protection against a virulent challenge in animal models^14^. Moreover, the same vector was successfully used in a DNA vaccine against canine leishmaniasis, encoding the activated protein kinase C receptor analog from *Leishmania infantum*. This vaccine showed effective protection in preclinical trials^15^ and was authorized for veterinary use by the European Commission on 20 December 2022, following a positive opinion from the European Medicines Agency’s Committee for Medicinal Products for Veterinary Use (CVMP). These results support the regulatory acceptability of the pPAL platform. In addition, our previous studies showed that a DNA vaccine targeting the Wuhan strain also provided protection against the Delta variant, likely due to the induction of potent cellular immunity and cross-reactive neutralizing antibodies. Building on this foundation, we designed a bivalent DNA vaccine using the pPAL platform that encodes spike proteins from two genetically distant SARS-CoV-2 variants, along with the nucleoprotein, to evaluate its immunogenicity and protective efficacy.

## Materials and methods

### Ethical statement

All the animal experiments were carried out in a disease-secure isolation facility level 4 according to the requirements of the WOAH (World Organization for Animal Health) at the Centro de Investigación en Sanidad Animal (CISA), in strict accordance with the recommendations in the guidelines of the Code for Methods and Welfare Considerations in Behavioral Research with Animals (EU Directive 2010/63 and Spain regulation RD53/2013, modified by RD1386/2018), and all efforts were made to minimize suffering. Experiments were approved by the CSIC Ethic Committee for Animal Experiments and the National Animal Welfare Committee (PROEX 295.6/21). All the study is reported in accordance with ARRIVE guidelines.

### pPAL plasmid construction and expression

We have constructed and used the pPAL mammalian expression plasmid vector which does not replicate in mammalian cells and does not contain selectable markers based on antibiotic resistance^13^. In addition to the previously described pPAL-Wu-Sfs expressing the stabilized Wuhan-H-1 spike and the pPAL-N expressing the nucleoprotein constructions^14^, a pPAL construct containing SARS-CoV-2 S protein-encoding gene from the omicron BA.1.1 subvariant (GenBank acc. no. ON394475.1) (pPAL-BA.1-Sfs) was obtained by gene synthesis (ATG Biosynthesis GmbH, Merzhausen, Germany) after codon sequence optimization for the human species by the Monte Carlo approach. The cleavage site modification to prevent furin cleavage (PRRA; PGGS; 681–684)^14^ was maintained for the omicron BA.1 spike construct, as well as a unique KpnI restriction site flanking the construct to remove the pGH plasmid vector in which the construct was generated. The pGH-pPAL-BA.1-Sfs was transferred to the *E. coli* SURE2 (Agilent, Santa Clara, CA) strain by electroporation at 1,800 V, 200 W, and 25 µF. Selection in LBagar medium was performed with 3 µM triclosan (Sigma-Aldrich, St. Louis, MO, United States). pGH was excised by KpnI (NEB, Ipswich, MA) digestion, and the vaccine constructs were circularized with T4 DNA ligase (NEB). Endotoxin-free pPAL, pPAL-Wu-Sfs, pPAL-BA.1-Sfs, and pPAL-N plasmid preparations were obtained with PureLink™ Expi Endotoxin-Free Giga Plasmid Purification Kit (Invitrogen, Waltham, MA) following the manufacturer instructions.

### Transfection of HEK293 cells and gene expression analysis

Cultures of HEK293 cells (CRL-1573™, ATCC^®^, Manassas, VA) were grown at 37 °C in a 5% CO_2_ atmosphere in DMEM supplemented with 10% heat inactivated fetal bovine serum, 100 IU penicillin–100 µg/mL streptomycin. The cells were detached and washed with sterile PBS and GTporator^®^-M (Protean, Ceske Budejovice, Czech Republic) per 3 million cells. Electroporation of 1.2 × 10^6^ cells with with 5 µg of pPAL-Wu-Sfs, pPAL-BA.1.-Sfs, or pPAL-N DNA was performed with an ECM 630 Electro Cell Manipulator Precision Plus^®^ (BTX, Cambridge, United Kingdom) at 220 V, 25 W, and 950 µF in 80 µL of GTporator^®^-M solution in a sterile 2 mm-gap cuvette (BTX, Cambridge, United Kingdom). Pre-warmed medium was immediately added to the cells, which were then placed in an 8-well culture slide (Nunc^®^ Lab-Tek^®^ Chamber Slide™, Sigma Aldrich, Burlington, MA) for indirect immunofluorescence assays and in a 24-well plate for gene expression analysis by Western blot. The cell suspensions were incubated at 37 °C for 24 h in a 5% CO_2_ atmosphere. Transfected HEK293 cells in 24-well plates were washed twice with 1 mL CM and lysed with 50 µL of a buffer containing 25 mM Tris-HCl pH 7.8, 2 mM EDTA, 2 mM DTT, 1% glycerol, and 1% Triton-X100. The protein extracts were quantified by the Bradford method. Each 20 µg protein extract was treated with 8.3 U/µL of TurboNuclease from *Serratia marcescens* (Sigma-Aldrich, Cambridge, United Kingdom) at room temperature for 10 min and prepared for SDS-PAGE in Laemmli buffer, heated at 95 °C for 5 min, and run at 30 mA for 90 min in 8–20% TGX precast SDS-PAGE gels (BioRad, Hercules, CA). Semi-dry transfer was performed on 0.45 μm nitrocellulose membranes (BioRad, Hercules, CA) at 1.3 A and 25 V for 10 min (high molecular weight transfer) in a TransBlot Turbo device (BioRad, Hercules, CA) following the manufacturer’s instructions. The membranes were blocked with 5% skimmed milk in PBS-0.1% Tween 20 (PBS-Tween) at room temperature under mild shaking for 1 h and washed thrice in PBS-Tween, 15, 5, and 5 min, respectively. The membrane was incubated at room temperature under mild shaking for 90 min with the primary antibodies prepared at the appropriate dilutions in blocking buffer. For spike protein detection, the primary antibody was a 1:800-diluted goat anti-S polyclonal antibody (Abcam #ab272504, Cambridge, United Kingdom), which only recognizes the S2 subunit. For nucleoprotein detection, the primary antibody was 1:500-diluted rabbit anti-N polyclonal antibody (kindly provided by Drs.M.Dominguez and I. Moreno, Instituto de Salud Carlos III, Madrid, Spain). The secondary antibody was 1:2000-diluted HRP-conjugated goat polyclonal anti-rabbit Ig.

For immunofluorescence studies, transfected cells in 8-well slides were washed once with 200 µL of a hypotonic solution (11:9 water: DMEM) and twice with 1:1 acetone: methanol. Fixation and permeabilization was performed with 1:1 acetone: methanol at – 20 °C for 10 min. The preparations were air-dried and the wells were carefully removed from the slides. Three 5 min washes with 0.22 μm filtered PBS were carried out in a Coplin jar. The preparations were then air-dried and blocked with 20 µL of a 5% skimmed milk solution in 0.22 μm-filtered PBS-Tween at 37 °C in a humid chamber for 1 h. After removing the excess blocking solution, 20 µL of a 1:50 dilution in blocking buffer of the anti-S2 and anti-N primary antibodies mentioned above were added to the corresponding preparation and incubated for 1 h. A single 5 min wash step was applied. Then, the cells were incubated with 20 µL of 1:200-diluted Alexa Fluor^®^ 488-conjugated goat antirabbit IgG secondary antibody (Jackson ImmunoResearch, West Grove, PA) at room temperature in the dark for 1 h. 10 min before the secondary antibody incubation was completed, 20 µL of 10 µg/mL DAPI in 0.22 μm-filtered PBS were added. The slides were washed four times with 0.22 μm-filtered PBS, 5 min per wash, and were then mounted with 50 µL Mowiol 4–88 and left to dry at 4 °C for 16 h in the dark. The fluorescence images were acquired with an SP8 STED 3× confocal microscope (Leica Wetzlar, Germany).

### Virus and virus stock preparation

SARS-CoV-2 viral strain Wuhan Hu-1 (MAD6) and the variant beta (B.1.351, Omicron), were kindly provided by Prof. Luis Enjuanes (CNB-CSIC, Madrid, Spain) and Dr. Juan García-Arriaza (CNB-CSIC, Madrid, Spain), respectively. These viruses were grown in the Calu3 cell line (kindly provided by Prof. Luis Enjuanes (CNB-CSIC, Madrid, Spain), propagated at a multiplicity of infection of 0.001 and stock obtained and tittered as described in^14^.

### Mouse immunization and challenge

8-week-old B6Cg-Tg(K18-hACE2)2Prlmn/J female mice (Charles River Laboratories, France) were inoculated with 80 µg pPAL-Wu-Sfs, 80 µg pPAL-BA.1-Sfs and 20 µg pPAL-N (pPAL-Sfs (Biv.) + pPAL-N) or with 180 µg pPAL as control using the PharmaJet modified Tropis NFIS (PharmaJet, Golden CO). The modified Tropis NFIS design is based on the regular Tropis^®^ device but it has been modified to deliver 50 µl injections at half the pressure of the regular Tropis device, making it suitable for injecting subcutaneously into mice. Briefly, the needle-free syringe was loaded with the adequate pPAL preparation and delivered subcutaneously under anesthesia with isoflurane into the gastrocnemius muscle. Three immunizations were performed at two-week intervals each. Animals (20 per group) were challenged with 10^5^pfu of SARS-CoV-2 Wu-H-1 (MAD6 isolate) two weeks after the last immunization. Animals were monitored daily thereafter, weighed and scored for clinical signs as described in^14^. Animals were sacrificed by cervical dislocation when they reached the humane endpoint as defined in the guidelines of the Code for Methods and Welfare Considerations in Behavioral Research with Animals EU Directive 2010/63 and Spain regulation RD53/2013, modified by RD1386/2018).

### Serum preparation

Blood for serum was obtained prior to immunization and challenge by submandibular venipuncture. Sera was obtained after allowing blood clotting at room temperature for 30 min followed by centrifugation at 10,000 × g for 5 min. Serum samples were stored at – 80 °C until use. For all experiments, sera were heat-inactivated at 56 °C for 30 min prior to use.

### Anti-RBD ELISA

ELISA were performed as described in^14^. Briefly, 96-flat bottom well ELISA plates (Costar) were coated with 200 ng/well recombinant RBD (Wuhan-H-1 or Omicron BA.1) (Raybiotech Life) overnight at 4 °C. Plates were then blocked with PBS + 0.1% Tween (PBS-T) + 3% BSA for 1 h at room temperature. After extensive washing in PBS-T, serial serum dilutions in PBS-T + 1% BSA were then added to the plates and incubated for 2 h at room temperature. Plates were then extensively washed with PBS-T, and incubated with protein-A-coupled to horseradish peroxidase (Thermofisher) to measure total Ig, or with anti-mouse IgG1-biotin (Biolegend), or with anti-mouse IgG2a/c-biotin (Biolegend) diluted in PBS-T + 1%BSA for 1h30min at room temperature. For IgG1 and IgG2a/c plates, an additional 1 h incubation step with streptavidin-HRP (Thermofisher) was performed after washing in PBS-T. Finally, after extensive washing in PBS-T, colorimetric reactions were developed using TMB substrate kit (Thermofisher) for 10–15 min and stopped with the addition of 2 N sulfuric acid prior to absorbance reading at 450 nm in a FLUOstar Omega plate reader (BMG Labtech). Antibody titers are presented as the dilution at which absorbance reading in pre-challenge samples is twice that of the 1:30 dilution of the naïve mouse serum.

### SARS-CoV-2 seroneutralization

These assays were performed as described in^14^. Briefly, serial dilutions of pre-challenge mouse serum were incubated with 100 plaque-forming units (PFU) or SARS-CoV-2 Wuhan-H-1 (MAD6) or Omicron BA.1 strain for 1 h at 37 °C in 96-flat-bottom well plates. Vero E6 cells (2 × 10^4^ per well) were then seeded and cultured in an incubator for 72 h at 37 °C, 5%CO_2_. Culture medium was then removed and cells fixed with 2% paraformaldehyde prior to staining with 2% crystal violet. Neutralizing antibodies (NAb) titers were defined as the dilution at which more than 50% of replicate wells did not display cytopathic effects.

### RNA extraction and RT-qPCR in target organs

To evaluate viral load in target organs, animals were sacrificed (5 per group) on day 4 and 7 post-infection (pi) and lungs and brain extracted. Organs were homogenized using a 2 min homogenization cycle in a tissueLyser II (Qiagen) at maximum frequency (30 Hz). Homogenate RNA was extracted using the IndiSpin Pathogen extraction kit (Indical) and stored at – 80 °C until use. Viral load was measured by RT-qPCR performed using the Luna Universal Probe One-step RT-qPCR Kit (New England Biolabs) using the primers and probes described in^14,16^.

### Statistical analysis and bioinformatics analysis

All statistical analyses were performed using the GraphPad Prism v8.0 software. Statistical tests employed for data analysis in experiments are indicated in the figure legends. Sequence alignments were performed with Clustal Omega and represented with Jalview 2.11.4.0. Structural modeling of proteins was carried out using AlphaFold 3.0., as reported^17^.

## Results

### Efficient expression of stabilized omicron spike protein using the pPAL platform

**Fig. 1:**
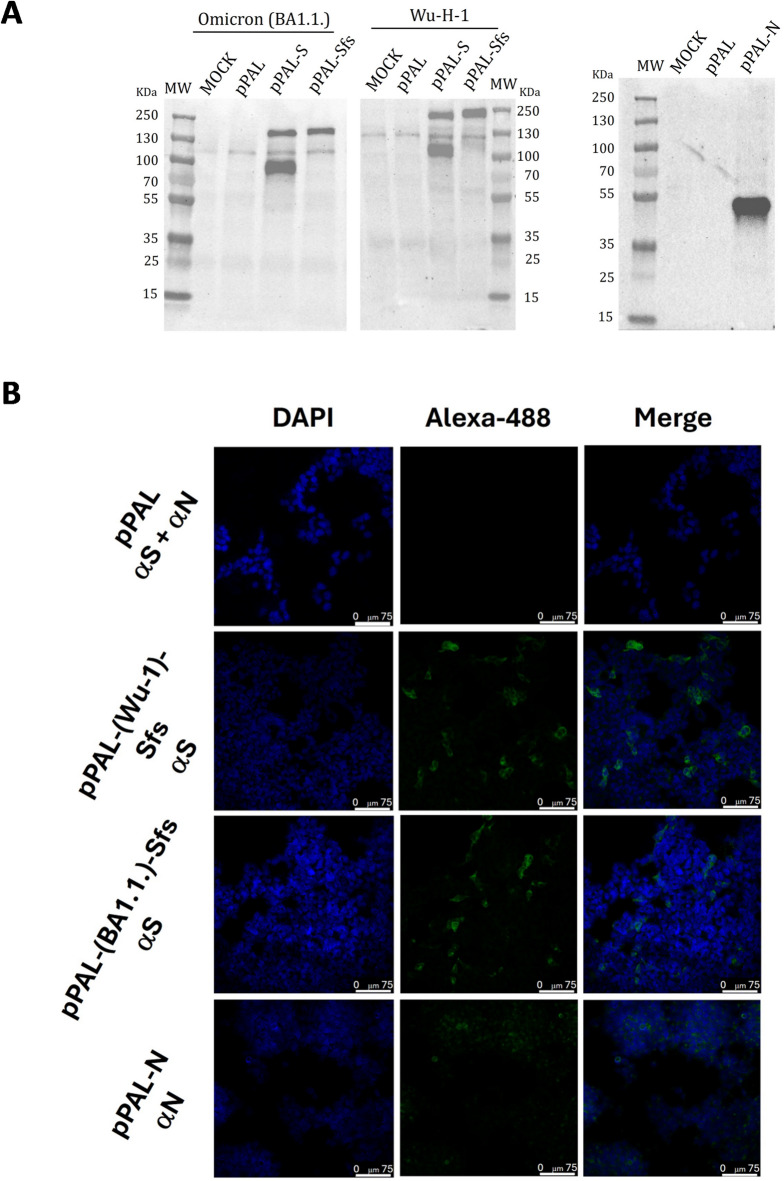
Stabilized fusion Spike (Sfs) protein expression using the pPAL vector. (**A**) (Left panel) Western blot of whole protein extracts from HEK293 cells transfected with pPAL, pPAL-S, and pPAL-Sfs obtained from Wuhan-H-1 and Omicron BA.1.1. strains. The primary antibody used for detection recognizes the S2 subunit, hence the detection of two bands with the non-stabilized S constructs that correspond to the native and processed spike protein. The stabilized Sfs constructs showed only one band at the predicted molecular weight, which confirmed that pPAL-BA.1-Sfs leads to the expression of a stabilized spike protein. (Right panel) Western blot of whole protein extracts from HEK293 cells transfected with pPAL and pPAL-N. (**B**) Immunofluorescence of cultured HEK293 cells transfected with pPAL, pPAL-Sfs (Wu H-1 and Omicron BA.1.1), and pPAL-N plasmids. The anti-S and anti-N antibody dilutions were 1:50. The pPAL control was incubated with both antibodies. The secondary antibody was Alexa Fluor^®^ 488-conjugated goat anti-rabbit IgG diluted to 1:200. Sfs from Wu H-1 and Omicron BA.1 as well as N expression is observed.

We aimed to develop a bivalent SARS-CoV-2 vaccine able of eliciting immune responses against divergent viral variants. To achieve this, we selected the stabilized spike protein (Sfs) from both the original Wuhan-H-1 strain and the distant Omicron BA.1 strain. The vaccine formulation also included the nucleoprotein (N), as its epitopes are relatively conserved across SARS-Cov-2 variants^18^, potentially promoting cross-variant cellular immune responses^19,20^. We had previously demonstrated successful expression of the Wuhan-H-1 Sfs and the N protein using our pPAL DNA vector platform^14^. Building on this, we cloned the Omicron BA.1.1 Sfs gene into the pPAL backbone. To asses expression, HEK293 cells were transfected with pPAL-Omicron.Sfs construct, and antigen expression was evaluated using Western blotting and immunofluorescence. As expected, the Omicron Sfs protein was sucessfully expressed and displayed a Western blot pattern nearly identical to that of the Wuhan-H-1 Sfs (Fig. 1A). Detection was performed using an antibody against the S2 subunit. For non-stabilized spike constructs, two bands were observed, corresponding to the native and processed spike forms. In contrast, the stabilized Sfs constructs yielded a single band at the expected molecular weight, confirming expression of the correctly folded, stabilized spike protein from pPAL-BA.1-Sfs. Expression of the N protein also produced a single band at the predicted molecular weight. Immunofluorescence analyses further confirmed expression of both Wuhan-H-1 and Omicron BA.1.1 Sfs proteins in transfected cells (Fig. 1B). The N protein was also clearly expressed, although the signal was less intense (Fig. 1B). Thus, pPAL construct encoding the Omicron Sfs efficiently expresses the stabilized spike protein, with a profile comparable to that of the Wuhan-H-1 Sfs construct, confirming tha platform’s capacity to express protective antigens from divergent SARS-Cov-2 variants.

### Bivalent pPAL-based DNA vaccine induces cross-reactive and Th1-biase humoral inmmunity in K18-hACE2 mice

**Fig. 2:**
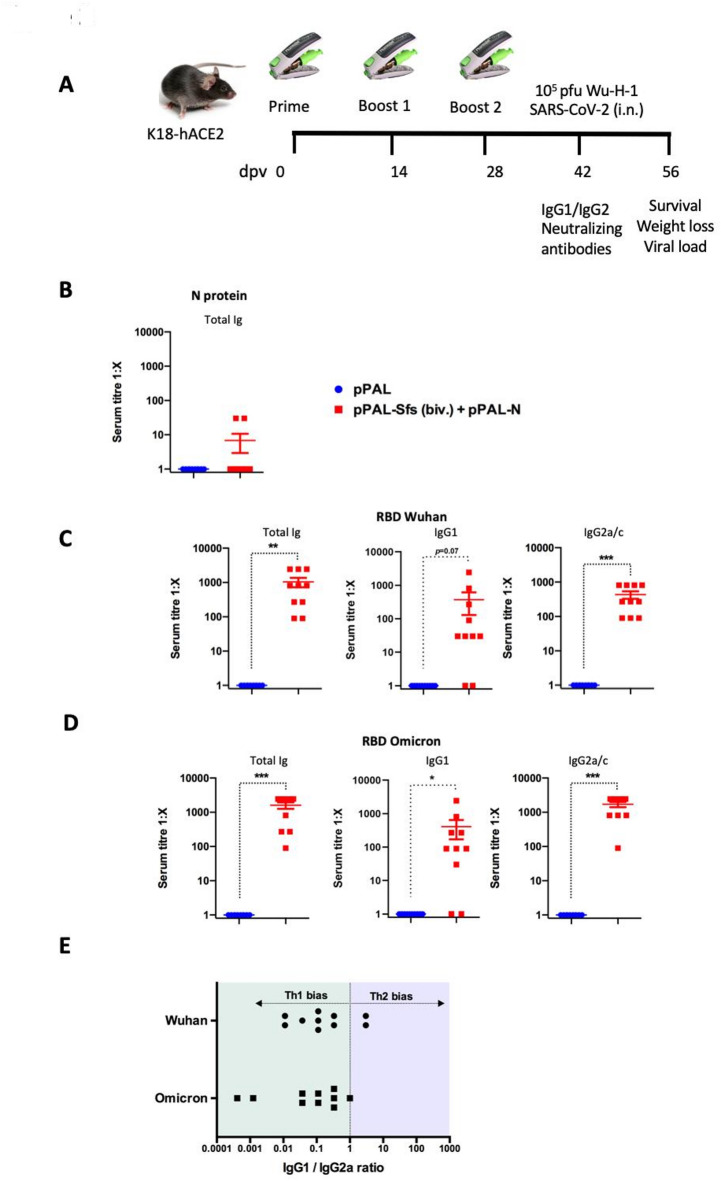
Bivalent pPAL vaccination induces antibodies to RBD from Wuhan and omicron variants. (**A**) Doses and immunization schedule for K18-hACE2 mice using vaccine candidates based on the spike protein from Wuhan-H-1 and omicron BA.1.1 strains of SARS-CoV-2. Vaccination was performed using the PharmaJet modified Tropis device, which deliver the vaccines at the subcutaneous tissue. In all cases, the spike protein used was the Sfs variant, a codon-optimized modified and modified version of the SARS-CoV-2 reference sequence designed to prevent furin cleavage of the protein product. Sera from immunized animals were collected prior to challenge and presence of antibodies recognizing the N protein, (**B**) Wuhan RBD (**C**) and Omicron RBD (**D**) was assessed by ELISA. Anti-N total Ig titre, (**B**) anti-Wuhan RBD (**C**) and anti-omicron RBD (**D**) total Ig, IgG1 and IgG2a/c titres are plotted. *p < 0.05; **p < 0.01; ***p < 0.001; Unpaired Student´s t-test. (**E**) IgG1/IgG2a titer ratio in sera of immunized mice.

To asses the humoral immune response elicited by the pPAL-based bivalent DNA vaccine (pPAL-Sfs (Biv.) + pPAL-N), K18-hACE2 mice were immunized using the modified Tropis needel-free injection system (NFIS) from PharmaJet, following schedule shown in Figure 2A. Mice injected with the empty pPAL vector served as placebo controls. Sera were collected after three inmunizations and prior to viral challenge. Antibody responses were measured by ELISA, targeting the recombinant N protein (Fig. 2B) and the RBD domain of the Wuhan (Fig. 2C) and Omicron (Fig. 2D) variants. Low levels of N-specific IgG were detected in two mice immunized with the bivalent vaccine. We then evaluated total IgG as well as the IgG1 and IgG2a/c isotypes specific to the RBD of both the Wuhan and Omicron strains (Fig. 2C,D). All vaccinated mice developed antibodies that recognize the RBDs from both variants, confirming the ability of the bivalent formulation to induce cross-reactive humoral immunity. Analyses of IgG isotypes revealed that most animals produced both IgG1 and IgG2a/c antibodies, with IgG2a/c titers consistently higher than those of IgG1. Calculation of the IgG1 to IgG2a/c titer ratios showed a consistent skew toward the IgG2a/c isotype (Fig. 2E), indicating a Th1-biased immune response, an outcome generally associated with effective antiviral immunity^21^.

### Bivalent pPAL vaccine elicitss neutralizing antibodies against Wuhan and Omicron variants

**Fig. 3:**
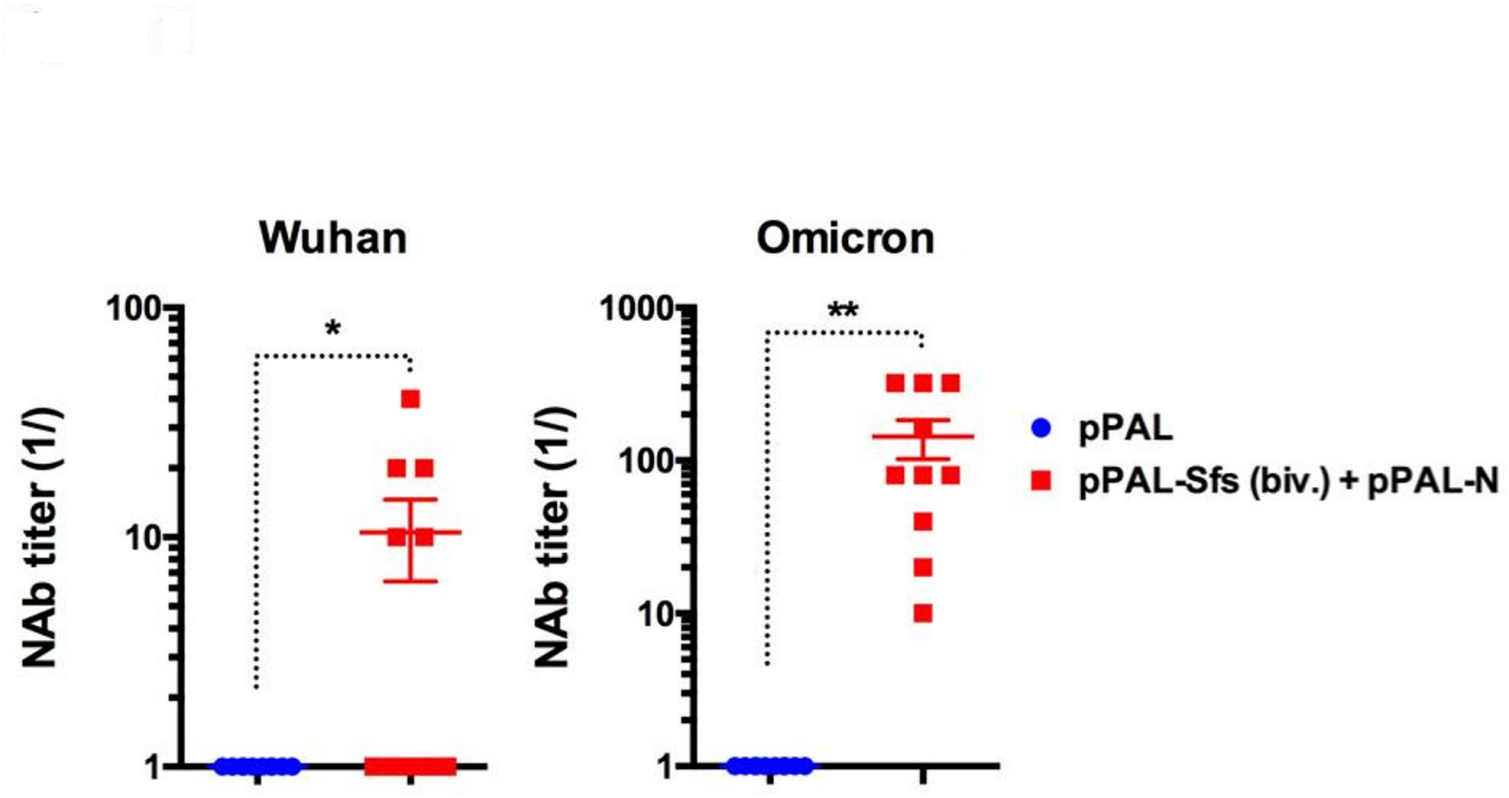
Bivalent pPAL vaccination induces neutralizing antibodies (NAb) to Wuhan and Omicron variants. Sera from immunized animals were collected prior to challenge and presence of neutralizing antibodies to Wuhan and Omicron variants assessed in neutralization assays. Serum dilutions were incubated with each variant and then co-cultured with Vero E6 cells to determine neutralization. Data are presented 1/the serum dilution (mean ± SEM) at which > 50% of cytopathic effect is blocked. *p < 0.05; **p < 0.01; Unpaired Student´s t-test.

We next assessed the ability of antibodies induced by the pPAL-Sfs (Biv.) + pPAL-N vaccine to neutralize SARS-Cov-2 infection in vitro (Fig. 3). All vaccinated mice developed neutralizing antibodies (NAbs) against the Omicron BA.1 variant. In contrast, NAb titers against the Wuhan-H-1 variant were lower, with only 50% of animals showing detectable levels above the threshold. These results demonstrate that bivalent pPAL vaccine can induce neutralizing antibodies against both variants, suggesting its potential to confer immunity against SARS-CoV-2 infection.

### Bivalent pPAL vaccination confers protection against SARS-CoV-2 Wuhan strain challenge

**Fig. 4:**
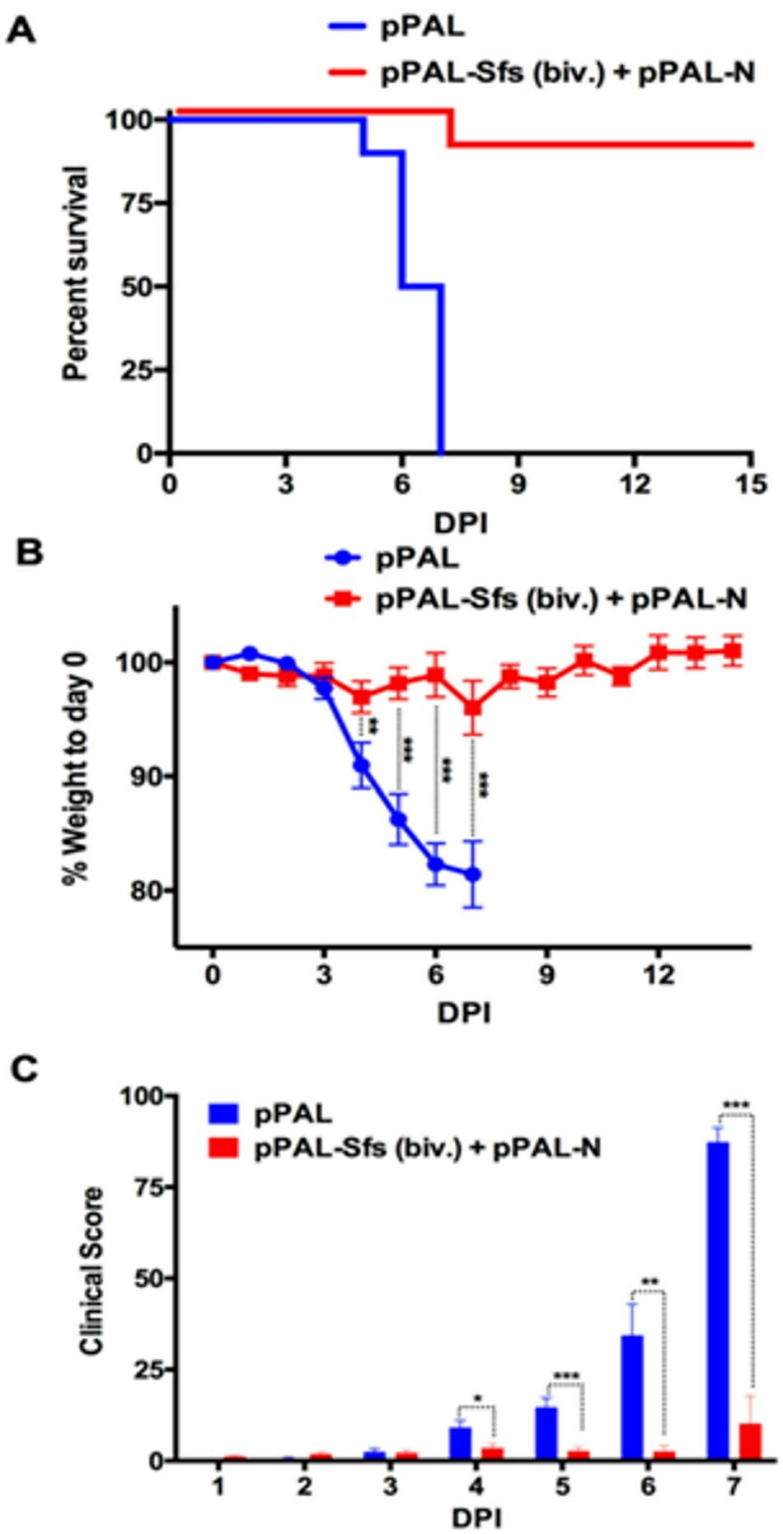
Bivalent pPAL vaccination protects against SARS-CoV-2 Wuhan strain challenge. Mice were challenged with 10^5^ pfu SARS-CoV-2 (MAD6) and weighted and monitored daily for clinical signs. (**A**) Survival of vaccinated pPAL-Sfs (biv.) + pPAL-N and control pPAL mice after challenge. (**B**) Weight loss in vaccinated and control mouse groups (mean ± SEM) after challenge. **p < 0.01; ***p < 0.001; Multiple t-tests with Holm-Sidak correction. (**C**) Clinical signs were scored in vaccinated and control mice after challenge. Mean ± SEM score for each group are plotted. *p < 0.05; **p < 0.01; ***p < 0.001; Multiple t-tests with Holm–Sidak correction.

To evaluate the protective efficacy of the pPAL-Sfs (Biv.) + pPAL-N vaccine, K18-hACE2 mice were challenged with the SARS-CoV-2 MAD6 (Wuhan-H-1) strain. Mice were monitored for survival and clinical signs (Fig. 4). Vaccination conferred 90% survival, whereas all control mice receiving the empty vector succumbed to infection by day 7pi. Body weight was tracked throughout the challenge, revealing that vaccinated mice experienced significantly less weight loss between days 4 to 7pi (Fig. 4B). Clinical scores were also significantly lower in vaccinated mice compared to controls over the same period (Fig. 4C), further confirming protection.

**Fig. 5:**
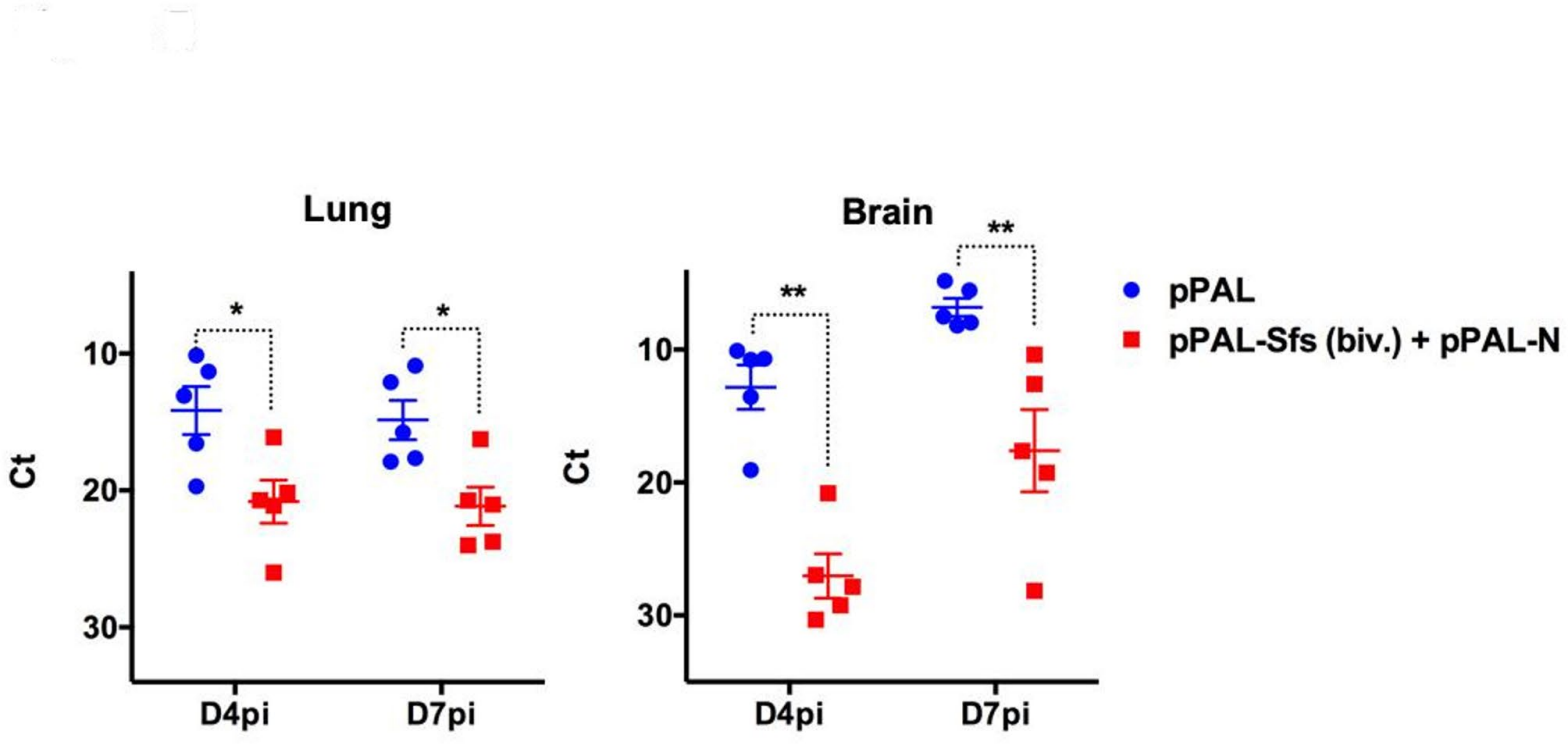
Bivalent pPAL vaccination reduced the SARS-CoV-2 load in target organs of infection. RNA was extracted from lung and brain of mice immunized with pPAL or pPAL-Sfs (biv.) + pPAL-N at day 4 and 7 post-infection (D4pi and D7pi) and viral load assessed by RT-qPCR. *p < 0.05; **p < 0.01; Two-way ANOVA with Fisher‘s LSD post test.

To asses viral replication, viral load was quantified in the lungs and brain, two key target organs in the K18-hACE2 murine model, using RT-qPCR in tissue homogenates collected at days 4 and 7pi in (Fig. 5). Vccinated mice showed a significant reduction in vira load in both the lungs and brain compared to controls. Importantly, viral spread to the brain a major contribution to disease severity in this model^22,23^, was notably diminished in vaccinated mice. These findings indicate that pPAL-Sfs (Biv.) + pPAL-N vaccination effectively limits viral replication in critical organs and provides robust protection against SARS-Cov-2-induced disease.

## Discussion

In this work, we present a bivalent SARS-CoV-2 vaccine based on the pPAL plasmid, a non-replicative antibiotic resistance gene-free expression vector. The global development of SARS-CoV-2 vaccines has progressed rapidly across various platforms, each with unique advantages. mRNA vaccines (Pfizer-BioNTech, Moderna) offer high efficacy and rapid adaptability to emerging variants, though they require ultra-cold storage. Viral vector vaccines (AstraZeneca, Johnson & Johnson) are more stables and easier to distribute but may lose effectiveness due to immune responses against the vector. Protein subunit vaccines (Novavax) are safe and stable, while inactivated virus vaccines (Sinovac, Sinopharm) rely on traditional methods but may elicit broader, less targeted responses. In this landscape, the pPAL-Sfs + pPAL-N DNA vaccine stands out for its safety, free of antibiotic resistance genes, and its ability to induce a strong Th1-skewed cellular immune response, which is critical for SARS-Cov-2 infection. Its thermostability, scalable production, and ease of adaptation to emerging variants make it a flexible and promising candidate for future pandemic preparedness.

Given the ongoing emergence of new variants, multivalent vaccines are increasingly necessary. DNA vaccines offer a strategic advantage in this context, as they can be easily modified to match evolving antigenic profiles of the SARS-CoV-2 spike protein. Aditionally, their production can be quickly scaled to meet global demand. Nonetheless, DNA vaccines require effective delivery methods to ensure optimal performance. In previous studies, we used electroporation as a proof of concept and demonstrated that expression of the spike protein and the nucleoprotein could protect mice against SARS-CoV-2 challenge^14^. Despite its effectiveness, electroporation can be painful for patients^24–26^, potentially limiting its acceptance. To address this, we opted to deliver our DNA vaccine using a needle-free injection system, an approach that has already been used in clinical trials for COVID-19 vaccination^27,28^. This method not only enhances patient confort but also eliminates the risk associated with needle use.

As a proof of principle for a bivalent vaccine, we designed a vaccine formulation that included two antigenically distant SARS-CoV-2 spike variants: the original Wuhan spike and the Omicron spike. To enhance their stability and immunogenicity, we mutated the furin cleavage sites in both spike proteins^14^, a modification known to improve immune responses^29^. Aditionally, we incorporated the nucleoprotein (N) to stimulate cellular immunity, aiming for cross-reactive T cell responses acroos different variants^20^. Initially, we evaluated multiple vaccine formulations, both with and without the inclusion of the N gene. Our results showed that only the combination of pPAL-Sfs and pPAL-N induced a strong cellular immune response characterized by high levels of IFN-γ-producing cells. This combined formulation previously demonstrated full protection in K18-hACE2 mice when administered via intramuscular injection followed by in vivo electroporation^14^. In the present study, we have further optimized the delivery method by using needle-free inoculation, a less invasive approach, while maintaining protective efficacy against the Omicron BA.1.1 variant. Following vaccination with pPAL-Sfs (Biv.) + pPAL-N combination, we detected only low titers of N antibodies in two mice. This limited humoral response to N could be due to immunodominance, where the more immunogenic spike proteins outcompete the nucleoprotein in eliciting antibody responses. It is worth noting, however, that antibodies against the nucleoprotein are readily detectable following natural infection^30^, confirming its inherent immunogenic. Another possible explanation is that nucleoprotein expression from pPAL occurs intracellularly, in contrast to the membrane-bound spike protein. This intracellular localization may preferentially engage T cell responses rather than antibody production, which aligns with our rationale for including the nucleoprotein in the formulation to promote robust, cross-reactive cellular immunity.

We found that vaccination with pPAL-Sfs (Biv.) + pPAL-N elicited anti-RBD antibodies against both spike variants included in the formulation. Furthermore, analyses of the IgG isotypes revealed a bias towards Th1-type immune response. This Th1skewing is desirable in the context of antiviral immunity, as it promotes the induction of effector mechanisms capable of celaring infected cells, including the activation of cytotoxic T cells or the promotion of antibody-dependent cell cytotoxicity (ADCC). This last mechanism is a particular relevant mechanism in the immune defense against enveloped viruses, as it targets viral proteins presented on the surface of infected cells^31–34^. In the case of SARS-CoV-2, ADCC has been identified as a correlate of protective immunity^35,36^. This process is mediated by Fc receptor-expressing effector cells, such as natural killer (NK) cells and neutrophils, which recognize antibodies bound to viral proteins on infected cells and initiate cell lysis. Indeed, in mice IgG2a are more efficient than IgG1 at triggering ADCC. In our study, we observed higher IgG2a than IgG1 titers, following vaccination, further supporting the conclusion that the pPAL-Sfs (Biv.) + pPAL-N vaccine induces a Th1-biased immune response and promotes effective immune recognition of SARS-CoV-2.

Vaccination with pPAL-Sfs (Biv.) + pPAL-N elicited neutralizing antibodies (NAbs) to both the Wuhan WuH-1 and Omicron BA.1 variants of SARS-CoV-2. The presence of NAbs is a well-established correlate of protection in SARS-CoV-2 infections^37,38^, indicating that our bivalent vaccine can induce immunity against two antigenically distant SARS-CoV-2 variants. Notably, we previously demonstrated that expression of the Wuhan Sfs using the pPAL system conferred protect against the Delta variant^14^, suggesting that this platform may provide broad spectrum protection against multiple SARS-CoV-2 variants. However, it is important to highlight that, in the current experiment, one mouse succumbed to infection, whereas in previous studies using monovalent vaccine, all animals survived the viral challenge^14^. Aditionally, we observed that NAb titers against the Wuhan strain were lower than those against Omicron. Although half of the immunized mice did not generate detectable neutralizing antibodies against the Wuhan-H-1 strain (Fig. 3), all vaccinated animals were fully protected (Fig. 4). This outcome highlights that antibody-mediated neutralization is not the sole mechanism of protection. In our previous study^14^ we demonstrated that immunization with pPAL-Sfs (Wu-H-1) + pPAL-N via intramuscular injection followed by in vivo electroporation elicited a strong Th1-type immune response and activated polyfunctional CD8^+^ T cells. These cellular responses are known to play a critical role in viral clearance and long-term immunity. Therefore, the observed protection in the absence of neutralizing antibodies can be attributed to the robust T cell-mediated immunity induced by the vaccine formulation. Furthermore, co-expression of multiple spike variants in a single vaccine may limit the immune response to individual components, potentially reducing protection against certain strains. This imbalance in the antibody response could be due to immunodominant epitopes within Omicron spike protein that preferentially drive the immune response. Alternatively, differential expression levels of the spike protein variants may influence the magnitude of the antibody response. Whether the observed disparity in NAb titers is due to differences in protein expression or intrinsic immunogenicity in the K18-hACE2 mouse model remains to be determined. Further work will be necessary and overcome this limitation in multivalent vaccine design.

Our data demonstrated that the bivalent pPAL vaccine provides protection against the Wuhan-H-1 isolate MAD6. Although needle-free inoculation is generally less immunogenic than electroporation, and only 50% of vaccinated mice developed NAbs against the Wuhan-H-1 strain, the clinical outcomes (survival, weight loss, and clinical score) were comparable to those obtained using electroporation-based vaccination^14^. These findings suggest that cellular immunity may contribute to protection in the absence of strong neutralizing antibody responses in this model. We were unable to evaluate protection against the Omicron BA.1 isolate, as K18-hACE2 mice are less susceptible to the disease caused by this variant^39,40^. However, vaccination induced significantly higher NAb titers against Omicron compared to Wuhan strain. Since NAb levels often correlate with protection, this raises the possibility that our vaccine may also be effective against Omicron. Although the K18-hACE2 mouse model was suitable for evaluating protection in this study, it has limitations in reproducing severe disease caused by Omicron variants. To further assess the cross-protective efficacy of the bivalent vaccine, future studies will be conducted using the Syrian golden hamster model, which is more susceptible to Omicron infection and better suited for evaluating clinical outcomes and viral clearance^41^.

Vaccinated mice also showed a marked reduction in viral load in the lungs, indicating effective control of viral replication in this primary target. Importantly, viral load in the brain was significantly lower in vaccinated animlas, suggesting that he vaccine helps limit viral dissemination to secondary organs like the brain, which are involved in the pathogenesis of severe disease^42^. Previous studies have shown that limiting brain infection is critical for a successful protective response^43^. Overall, our bivalent vaccine appears to control viral replication effectively, resulting in protection from disease in this model.

In this study, we present a proof-of-concept bivalent DNA vaccine for SARS-CoV-2 that lacks antibiotic resistance genes for selection during production. This DNA construct allows for the expression of spike proteins from two distint variants, inducing NAbs against both. The vaccine elicits a Th1-biased immune response, which is optimal for antiviral protection. It also confers protection against a virulent SARS-CoV-2 challenge, reducing clinical signs and viral replication in key target organs. Given the ease of modifying DNA vaccines to express new isoforms of the spike protein and their scalability in production, the use of the pPAL backbone offers a promising platform for multivalent vaccination against emerging SARS-CoV-2 variants.

## Data Availability

Any additional information required to reanalyze the data reported in this work paper is available from the lead contact upon request to Noemí Sevilla (sevilla@inia.csic.es) or Jose M. Rojas (rojas.jose@inia.csic.es).
